# *In vitro* cultivation of adherent cells on microscope slides for downstream applications

**DOI:** 10.1016/j.mex.2024.102847

**Published:** 2024-07-05

**Authors:** Kine Samset Hoem, Hanne Lillerovde Ørstenvik, Anne Elin Varhaugvik, Ann-Kristin Tveten

**Affiliations:** Department of Biological Sciences Aalesund, Faculty of Natural Sciences, Norwegian University of Science and Technology, Larsgardsvegen 2, Aalesund 6009, Norway

**Keywords:** Cell culture, Microscopy, A549, RTgill-W1;ASK, Histological staining, Gram stain, Alcian blue, Haematoxylin-eosin, Effective cell cultivation on microscope slides

## Abstract

In vitro studies with cultured cells are often conducted as an important part of basic research. Adherent cells are typically cultivated in flasks or trays, for which cell staining and subsequent visualization become impractical. We here present a simple step-by-step method for growing adherent cells directly on glass microscope slides, using low-cost equipment readily available in most laboratories. Most parameters such as type of microscope slide (e.g. surface coating), cell seeding concentrations and incubation times can be adjusted according to cell line characteristics and experimental aims, reflecting the methods’ flexibility. Through our experiments, microscope slides proved to provide an acceptable surface for cell adhesion and growth of the tested cell lines, as well as being robust and functional with respect to downstream procedures. The method can potentially be combined with different techniques for visualization of experimental effects, such as histological staining methods, fluorescent staining, and immunochemistry. In our method development we have successfully cultivated three different cell lines directly on microscope slides – Atlantic salmon kidney cells (ASK), rainbow trout gill cells (RTgill-W1), and human cancerous lung cells (A549) – and subjected them to various experimental treatments. Finally, as proof-of-concept we provide examples of successful histological staining of the fixed cells.

Experimental design in short:

• Cultivate cells and calculate cell concentration
• Seed a small volume of growth medium with an appropriate number of cells on microscope slide in an area confined by hydrophobic marker
• Let cells adhere over night before adding more growth medium or directly conducting experiments and fixing cells for downstream applications

## Graphical abstract

**Figure:**
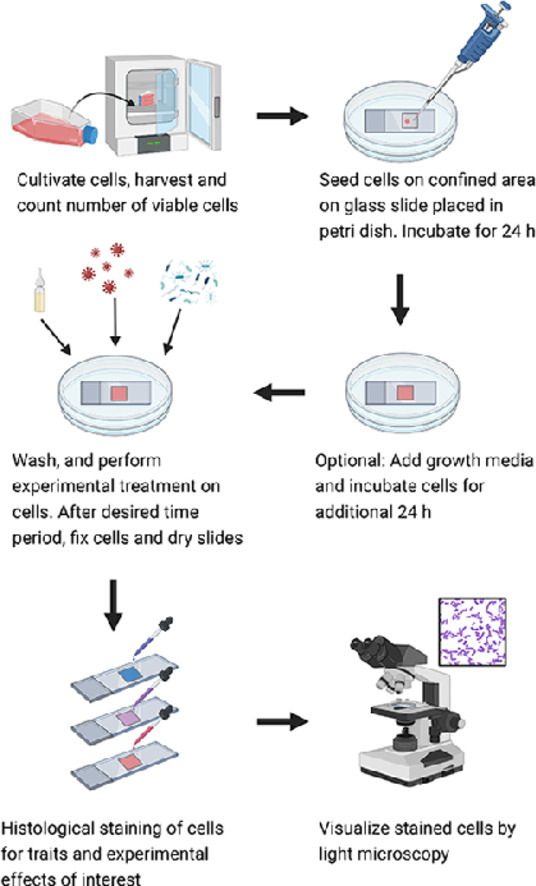
In vitro cultivation of adherent cells on microscope slides for downstream applications. The figure was created with BioRender.com.

Specifications table

**Table:** 

Subject area:	Biochemistry, Genetics and Molecular Biology
More specific subject area:	*In vitro* cultivation of adherent cells
Name of your method:	Effective cell cultivation on microscope slides
Name and reference of original method:	N.A.
Resource availability:	N.A.

## Method details

### Introduction

In vitro studies of cell lines may provide useful insight into cellular functions. Cells can be explored in several ways, including the use of fluorescent dyes or labels (e.g. green fluorescent protein), immunochemical techniques, or histological staining [[1], [2], [3]]. Visualization of experimental effects, cell structures and morphology can then be achieved through different forms of microscopy. Microscope images may also serve as a useful supplement to support other analyses, e.g., transcriptional studies, as addressed in Hoem and Tveten [4]. For instance, while reverse transcription quantitative PCR (RT-qPCR) analyses may demonstrate the presence of bacteria after an infection trial, histological (Gram) staining may elegantly support this implication by visualizing the presence of bacteria inside the cell cytoplasm.

Adherent cells are grown in cell flasks or trays. For studies with downstream evaluation under a microscope, these containers tend to be impractical, and an extra transfer of the cells to a fitting substrate may be problematic. Today, the most common alternatives for growing cells for microscopy purposes is the use of coverslips or chamber slides (e.g. [[5], [6], [7]]). Chamber slides can be expensive, and leakage and contamination can be a risk if not correctly handled. Coverslips are fragile and can be difficult to handle. They are typically placed in plate wells and added a cell suspension for cultivation. One problem with this setup is that cells can potentially start growing on both sides of the coverslip, as well as other surfaces in the well. As a result, the actual number of cells on the coverslip surface intended for microscopy may vary substantially. There is thus a need for a cost-effective and practical method for growing adherent cells on a surface compatible with downstream applications, without the need for future transfer.

In this article, we present a simple step-by-step method to cultivate adherent cells directly on microscope slides using equipment that is generally cost-effective and readily available in most laboratories. For instance, the cost of microscope slides and petri dishes combined may be under half the cost of specialized chamber slides, depending on surface material and coating [8]. While coverslips are also reasonably priced, there is a trade-off with the potential risks of use, as previously outlined. Moreover, the method does not require any prior expertise or specialized training by the users besides being trained in standard cell culture work. To our knowledge there is no openly available literature describing a similar approach.

The method bases itself on the use of microscope slides placed in individual petri dishes and a hydrophobic marker to confine a growth area for cells on the glass surface. In this way, cells can be directly fixed and stained in place without disruption of the cell monolayer. In our method development we have applied three different cell lines – ASK, RTgill-W1 and A549 – and different experimental protocols. We successfully stained slides using different histological techniques to demonstrate the method's potential usefulness. For the applied cell lines, microscope slides proved to provide an acceptable surface for cell adhesion and growth, as well as being robust and functional in terms of various downstream procedures.

### Workflow

**Step 1: Cell cultivation**

Cell lines of interest are first to be cultivated. In our method development, we applied the commercially available cell lines ASK (CRL-2747, ATCC), RT-GillW1 (CRL-2523, ATCC) and A549 (CCL-185, ATCC). ASK are Atlantic salmon (*Salmo salar*) kidney cells with a doubling time of 10–17 days. RTgill-W1 are gill cells from rainbow trout (*Oncorhynchus mykiss*) with a doubling time of 10–12 days, and A549 are cancerous human lung epithelial cells with a short doubling time of 1–2 days. Cell cultivation was conducted according to recommendations from ATCC [9]. ASK and RTgill-W1 were cultivated in Leibovitz's l-15 medium (HyClone), supplemented with 1% Antibiotic Antimycotic (AA, Gibco), and 20% and 10% fetal Bovine Serum (FBS, Gibco), respectively. Both salmonid cell lines were incubated at 20 °C, 100% air. A549 was grown in Dulbecco's Modified Eagle Medium F12 (DMEM/F12, ThermoFisher) with 10% FBS and 1% AA in 37 °C, 5% CO_2_.

**Step 2: Seeding cells on microscope slides**

Sterile equipment and conditions must be ensured in all steps prior to cell fixation. In this step, cells are seeded onto microscopy slides in a way that ensures a dense monolayer within a confined area. Before seeding for experiments, cell counting is a necessary step to confirm proliferation of cells and to calculate cell concentration. A number of cell counting methods are available. Adjust cell number according to cell type and growth (see “Method validation” section).

**Fig. 1:**
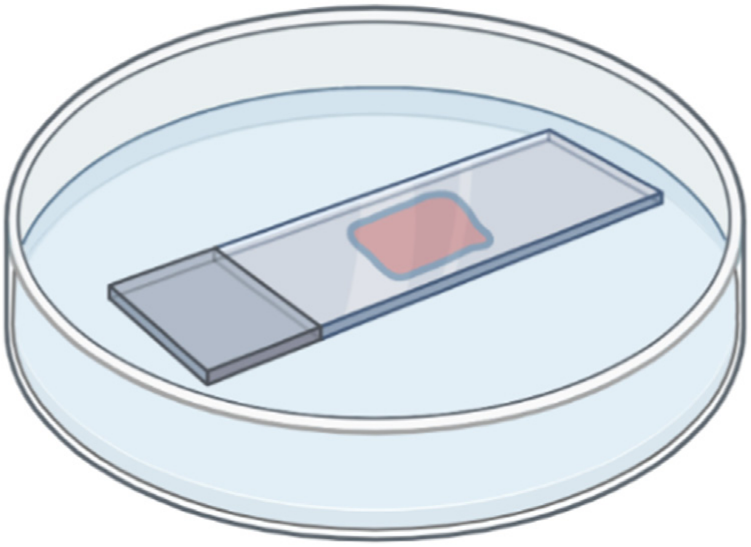
Material setup. Microscope slide containing cells in a confined area, placed in petri dish. The figure was created with BioRender.com.
  5. After incubation overnight the cells can either be supplied with growth medium (20 ml) in the petri dish for further cultivation or go directly on to experimental treatment. The latter is dependant on cell status, cell line characteristics, and experimental design.

Materials and reagents:

• Cell suspension
• Microscope slides
• Pencil
• Petri dishes with lid (diameter large enough to fit glass slide)
• Hydrophobic marker
• Pipette (100–1000 µl)
• Pipette tips
• Serological pipette
• Growth medium
  1. Microscope slides (Superfrost, Epredia) were labelled with pencil and placed in petri dishes with lid.
  2. Hydrophobic marker (Dako pen (Dako S2002)) was used to confine the area of cell growth on slides, approximately 2 cm x 2 cm (4 cm^2^).
  3. Cell suspension (400 µl) was carefully applied onto the confined area on slide. We found this volume to be appropriate for our experiments as it did neither evaporate overnight nor overflow the hydrophobic boarders – however, the volume could potentially be adjusted. We also found it favourable to place the pipette tip against the glass surface when dispensing the cell solution instead of dripping it, as this placed less mechanical stress on the hydrophobic boarder. NB: Cell suspension was thoroughly homogenized before each pipetting, aiming to seed an equal number of cells to all slides.
  4. Cells were left to set in incubator for 24 h or overnight to ensure cell adhesion within the confined area only. Setup of slide in petri dish is illustrated in Fig. 1.

**Step 3: Experimental treatment**

Adherent cells on microscope slides can be subjected to desired experimental treatments to study specific cellular responses. Such treatment could include viral agents, bacterial agents, drugs and chemicals, as well as other manipulations (e.g., siRNA-mediated gene knockdown). We emphasize that the method can be tailored significantly according to research aims, and the following sections are only examples of what could be done. For our method testing we conducted a virus infection trial, a coinfection trial with bacteria and virus, and a trial to assess the effects of a chemical substance.

A. **Infection trial with virus**

ASK cells (1000 000 cells/ml) were seeded onto microscope slides as described. In this case, the experimental treatment was initiated directly after an incubation time of 24 h (point 5 in Step 2). Cells were then subjected to infectious salmon anaemia virus (ISAV, VR-1554, ATCC) for 1 hour or continuously throughout a time series. We conducted the following steps:

1. The growth media in the area (4 cm^2^) confined by hydrophobic marker was carefully removed by pipette.
2. ISAV (2 x TCID_50_, 400 µl) was added to the confined area on microscope slides for experimental samples. Control cells were added growth medium. Samples intended for continuous virus exposure were incubated according to point 6 below.
3. Samples intended for short virus exposure were incubated with ISAV for 1 hour (15 °C, optimal ISAV replication temperature) to allow infection to take place.
4. Cells were washed twice by carefully rinsing DPBS (3 ml) over the microscope slides.
5. Petri dishes were added fresh growth medium (20 ml).
6. Cells were incubated in 15 °C for 6, 24 and 48 h to evaluate cellular responses to short and continuous virus exposure.

B. **Coinfection trial with bacteria and virus**

A549 cells were used in a coinfection trial including human enterovirus D68 (EVD68, VR-1197, ATCC) and group A strep *Streptococcus pyogenes* (GAS, 19,615, ATCC). Cells were seeded onto microscope slides (500 000 cells/ml). Experimental treatment was initiated directly after an incubation time of 24 h (point 5 in Step 2). We conducted the following steps:

1. Viral agent EVD68 (2.5 x TCID_50_) and bacterial agent GAS (2.5 MOI) were prepared as homogenic solutions in culture media.
2. The growth media in the area (4 cm^2^) confined by hydrophobic marker was carefully removed by pipette. Control cells were added growth medium.
3. Cocktails with viral and bacterial agents (400 µl) were added the cells.
4. Petri dishes were incubated for 2 h (37 °C) to allow infection to take place.
5. Cells were washed twice by carefully rinsing DPBS (3 ml) over the microscope slides.
6. Petri dishes were carefully added fresh growth medium (20 ml).
7. Cells were incubated (37 °C) to evaluate results of coinfection with EVD68 and GAS after 12, 24 and 36 h.

C. **Chemical substance trial**

RTgill-W1 cells (1400 000 cells/ml) were seeded onto microscope slides as previously described and incubated for a total of 48 h before experimental treatment. Cells were then exposed to a substance of interest: N-acetylneuraminic acid (Accobio, CAS number 131–48–6). Chemicals can be directly added to the growth medium in the petri dishes, however, to ensure homogeneity in the solution we suggest the following steps:

1. The chemical was first prepared in different concentrations of interest in growth medium.
2. All growth medium in the petri dishes was carefully removed by pipette.
3. N-acetylneuraminic acid (0.4 mM, 400 µl) was added to the area (2 × 2 cm^2^) previously confined by hydrophobic marker. Control cells were added growth medium.
4. Petri dishes with microscope slides were incubated (20 °C) for 1 hour to allow the chemical to exert effect on the cells.
5. Cells were washed twice by carefully rinsing DPBS (3 ml) over the microscope slides.
6. Petri dishes were added fresh growth medium (20 ml).
7. Cells were further incubated (20 °C) to evaluate cellular effects of the chemical after 24 h.

**Step 4: Fixing cells on microscope slides**

After concluding experimental treatments, cells were fixed to the glass prior to staining and microscopy. Fixation immobilizes the cellular processes and maintains the integrity and organization of the cell monolayer. Depending on downstream applications, fixed cells can potentially be stored for days or weeks before moving on with the procedure.

Materials and reagents:

• Serological pipette
• Scalpel blade
• Dulbecco's Phosphate Buffered Saline (DPBS)
• Fixation spray
• Tissue paper
  1. All medium was removed from the petri dishes holding the slides.
  2. Cells were washed twice by carefully rinsing DBPS (3 ml) over the cells.
  3. A scalpel blade was used to lift each slide to remove it from the petri dish. The slides were placed on tissue paper to airdry lightly.
  4. When the slides were nearly dry, they were fixed by the use of fixation spray (Merckofix spray fixative, Merck).

**Step 5: Histological staining**

One benefit with the developed protocol is the ability to perform histological staining directly on adherent cells fixed on microscope slides. The general outline of histological staining involves: a) hydration, b) staining, c) dehydration, d) clearing and e) cover-slipping. Depending on area of interest different types of staining techniques may be applied. As proof-of-concept in the study we have performed histological staining with three common staining protocols on the different cell lines: haematoxylin-eosin (HE), alcian blue, and Gram stain. The HE-stain will highlight different cellular components, while alcian blue can be used to visualize acidic mucins [10]. Gram stain will colour bacteria based on their cell wall structure. Bacteria with cell walls rich in peptidoglycan, so-called Gram positive, will appear violet/blue after the staining process, while Gram negative bacteria (as well as eukaryotic cells) will appear pink/red [11].

A. **Haematoxylin-eosin (HE) stain:**

ASK cells were stained with HE to evaluate early morphological effects of infectious salmon anaemia virus infection.

1. Fixed cells were treated with decreasing concentration of ethanol, from 100% EtOH to 95% EtOH and then 70% EtOH before transferring microscope slides to distilled water. Microscope slides were dipped into each solution approximately 10 times with a slow movement.
2. Microscope slides were transferred to Harris’ haematoxylin solution and incubated for 3 min.
3. Bluing of cells was performed in water (temperature approximately 35–37 °C) for 5 min. The haematoxylin now changes colour from red to blue.
4. Microscope slides were transferred to erythrosine (1%) for 1 min.
5. Microscope slides were rinsed in water by 10 slow dips in a clean water bath.
6. Dyed slides were dehydrated in increasing concentration of ethanol, from 70% EtOH via 95% EtOH and finally 100% EtOH for about 10 slow dips in each solution.
7. Finally, the microscope slides were treated with xylene before mounting of cover slips.

B. **Gram stain**:

A549 cells that had been coinfected with human enterovirus D68 and the Gram positive bacteria *Streptococcus pyogenes* were Gram stained to evaluate whether the degree of bacterial infection was affected by the viral presence.

1. Fixed cells were treated with decreasing concentration of ethanol, from 100% EtOH to 95% EtOH and then 70% EtOH before transferring microscope slides to distilled water. Microscope slides were dipped into each solution approximately 10 times with a slow movement.
2. Microscope slides were transferred to crystal violet solution, incubated for 30 s, and then rinsed in water with 5–10 dips.
3. Microscope slides were transferred to Weigert's iodine solution, incubated for 20 s, and then rinsed in water with 5–10 dips.
4. Decolouring of microscope was conducted in acetone for 5–10 s, and then rinsed in water with 5–10 dips.
5. Microscope slides were transferred to 0,5% core red solution, incubated for 3 min, and then rinsed in water with 5–10 dips.
6. Filter paper was used to wipe the microscope slides dry. Slides were left to dry completely for 15 min in fume hood.
7. Microscope slides were further dehydrated in acetone for 5–10 dips.
8. Finally, the microscope slides were treated with xylene, before mounting of cover slips.

C. **Alcain blue stain**:

RTgill-W1 cells treated with N-acetylneuraminic acid were stained with alcian blue to evaluate whether the chemical affected the cells’ mucin production. Alcian blue is here combined with haematoxylin to further highlight cellular features.

1. Fixed cells were treated with decreasing concentration of ethanol, from 100% EtOH to 95% EtOH and then 70% EtOH before transferring microscope slides to distilled water. Microscope slides were dipped into each solution approximately 10 times with a slow movement.
2. Microscope slides were transferred to alcian blue solution and incubated for 10 min.
3. Microscope slides were rinsed in water with 5–10 dips.
4. Microscope slides were transferred to haematoxylin and incubated for 1 min.
5. Bluing of cells was performed in water (temperature approximately 35–37 °C) for 5 min. The haematoxylin now changes colour from red to blue.
6. Dyed slides were dehydrated in increasing concentration of ethanol, from 70% EtOH via 95% EtOH and finally 100% EtOH for about 10 slow dips in each solution.
7. Finally, the microscope slides were treated with xylene, before mounting of cover slips.

**Fig. 2:**
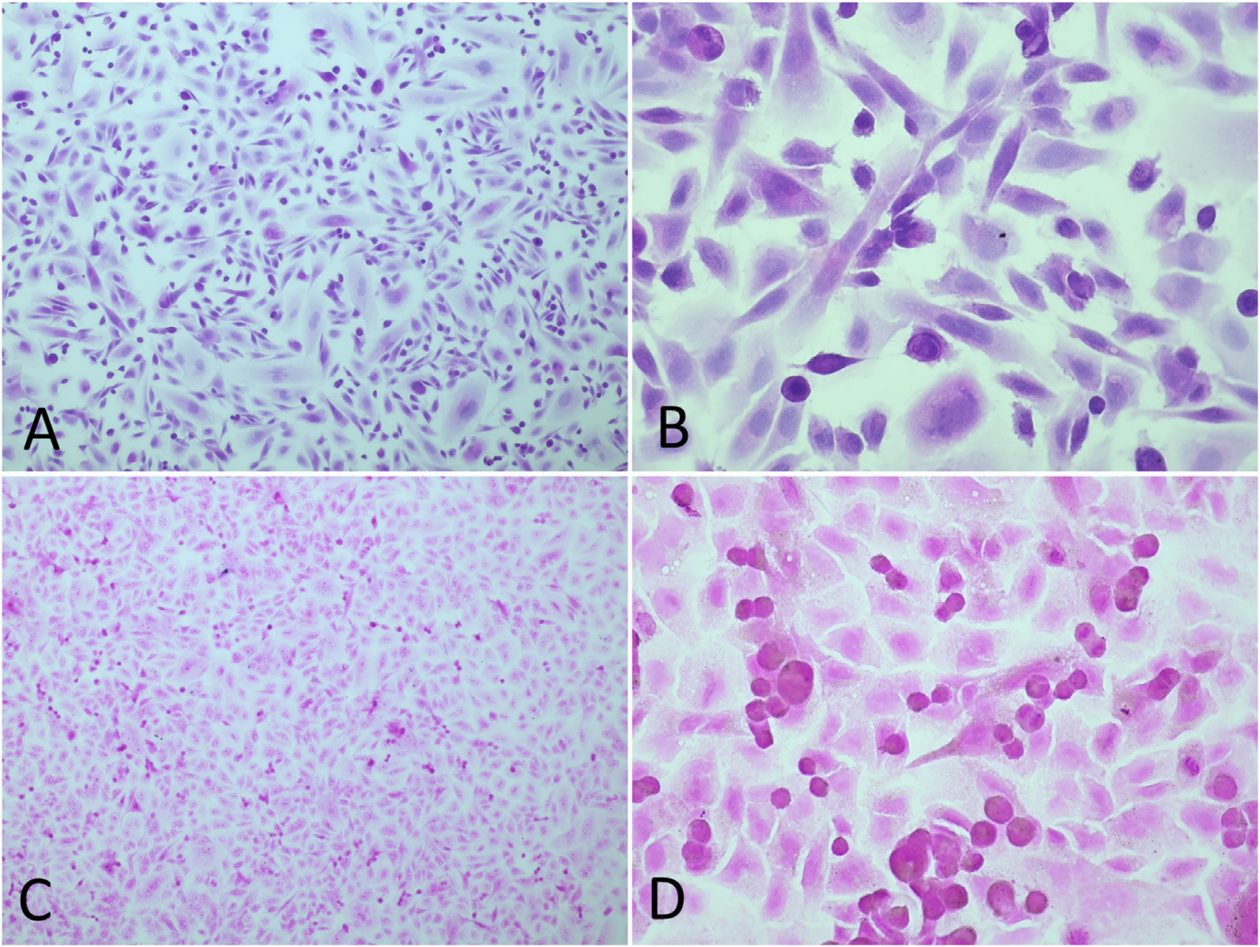
Example images of cells after histological staining. A and B: ASK cells stained with HE, 10x and 40x magnification, respectively. C and D: A549 cells stained with Gram stain. 10x and 40x magnification, respectively.

Depending on staining method, fixed and stained slides can be stored for longer periods of time without losing visual characteristics. Microscopy images included in this article were taken 6 weeks after staining took place. Fig. 2 represents an example of the type of images that can be obtained from histological staining of adherent cells and that could be included as supporting results in a publication.

## Method validation and general considerations

**Fig. 3:**
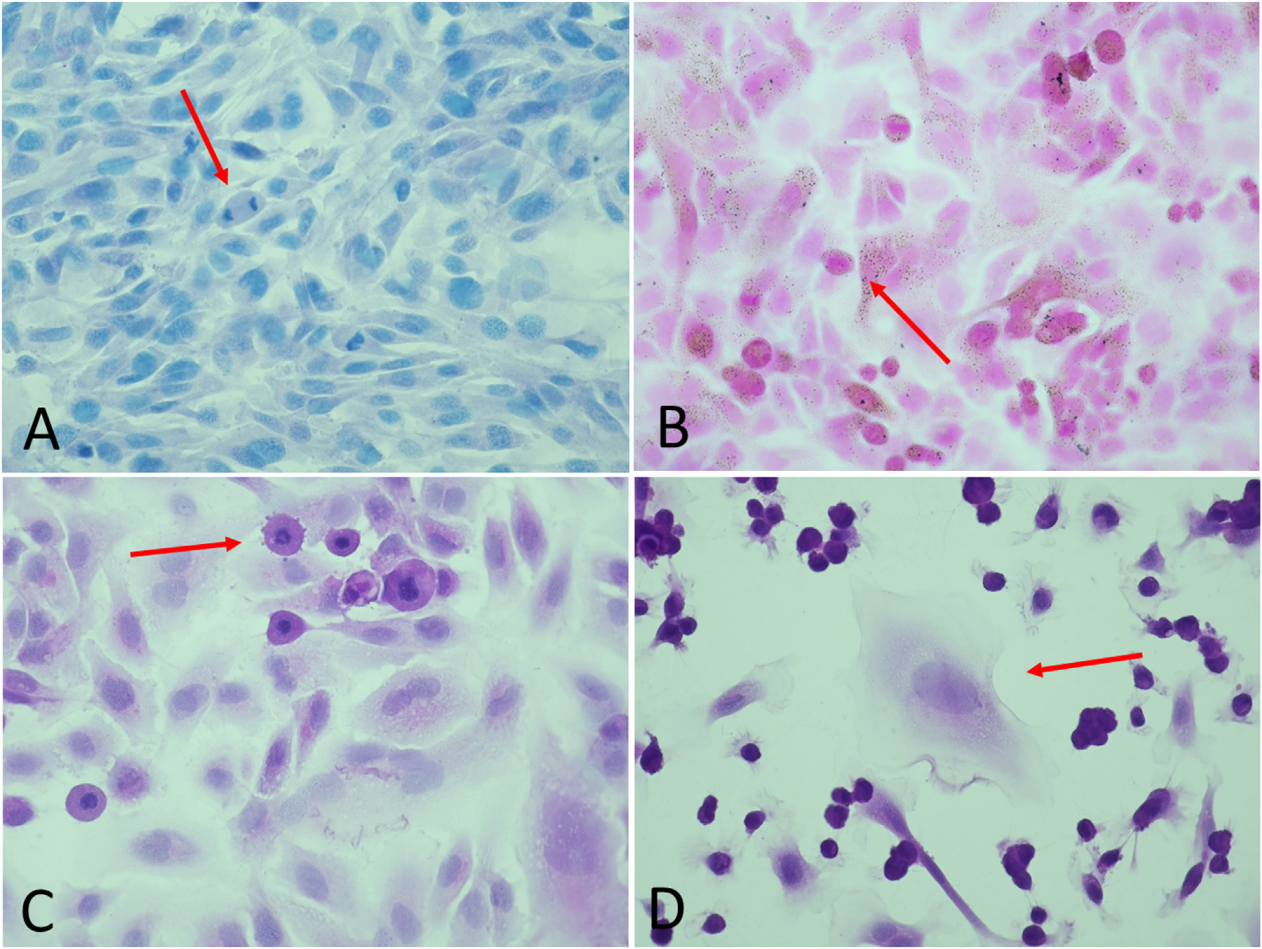
Examples of observable cellular characteristics and responses. A: Mitosis in RTgill-W1, stained with Alcian blue. B: Gram-positive (blue) GAS bacteria inside A549 cells, coloured with Gram stain. C: Apoptosis in ASK cell, stained with HE. D: Disruption of ASK cell, stained with HE. Magnification is 40x for images B, C and D and 10x for image A.

Frequent examination under a light microscope confirmed normal growth on the slides and that the hydrophobic boarders prevented cells from growing outside the marked area. When cell seeding concentration was appropriate, cells were visualized as a monolayer. This provides a useful starting point for cell experiments as well as appealing microscope images. The method was further validated through several microscopy observations. Fig. 3 provides concrete examples of visual characteristics of cellular behaviour and responses that can be observed with the described method and that can further support other research data. Proliferation on microscope slides was confirmed by the observation of cell division (mitosis), as illustrated in Fig. 3A. Despite several rounds of washing, cells still attached to the slide surface. Together these observations indicate that the microscope glass surface provided an acceptable surface for cell adhesion and normal growth for the applied cell lines ASK, RT-gillW1, and A549. Histological staining highlighted the presence of bacteria inside cell cytoplasm in Fig. 3B, and viral infection induced apoptosis and cell disruption, as illustrated in Fig. 3C and D, respectively.

**Fig. 4:**
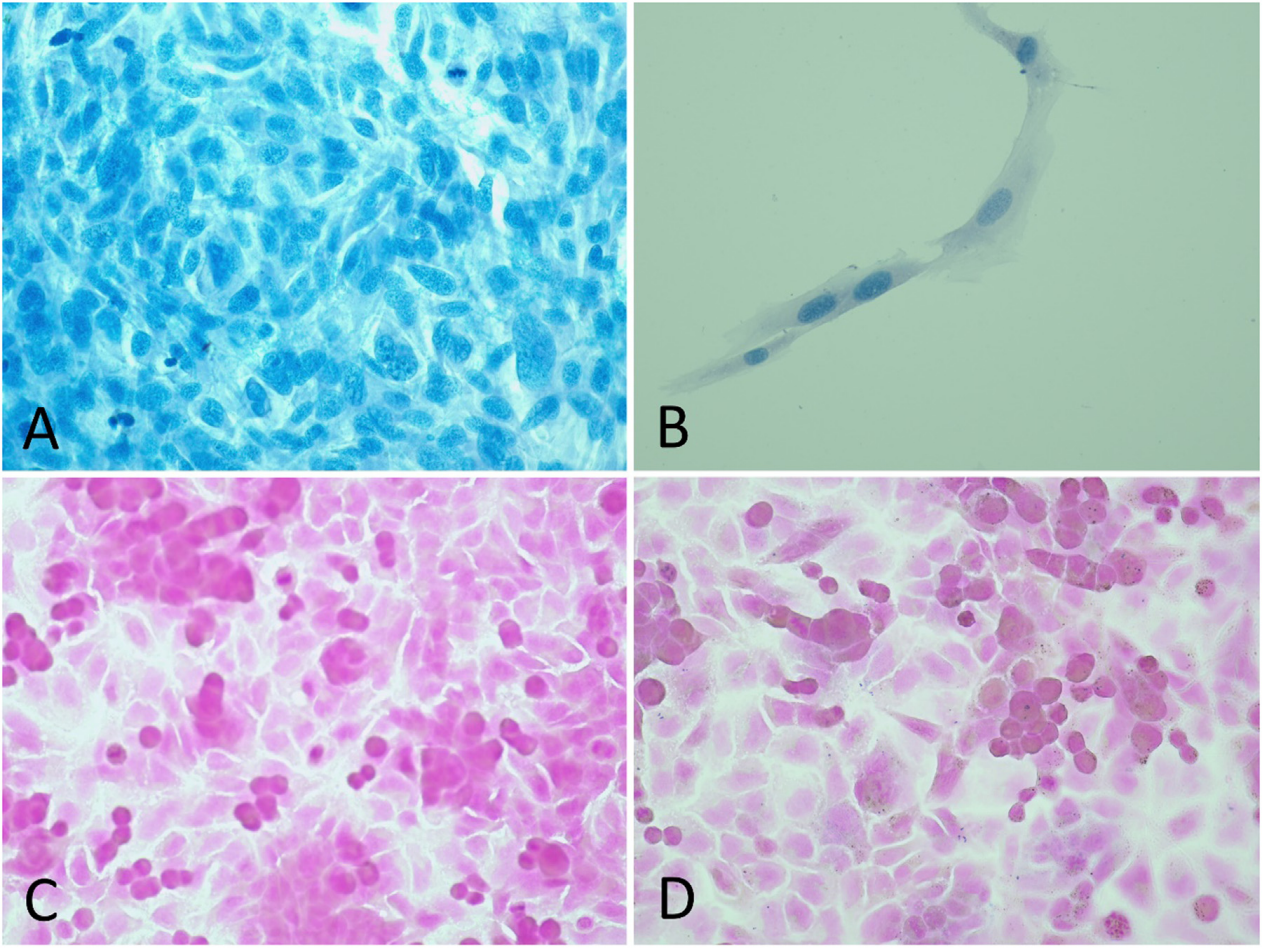
Examples of potential challenges in cell seeding and histological staining. A: RTgill-W1 cells seeded in too high density, stained with alcian blue, 40x magnification. B: RTgill-W1 cells seeded in too low density, stained with alcian blue, 40x magnification. C: Properly dehydrated A549 cells, strong pink colour from the Gram stain. D: Poor dehydration results in weaker colour and brownish areas on Gram stained A549 cells.

When setting up an experiment, it should be kept in mind that cell lines differ in growth rate as well as cell size. Therefore, in accordance with the cell line of interest and desired cell density, the number of cells seeded on the microscope slides must be adjusted. Cells should be visualized as a monolayer. In our case, we used a lower concentration (500 000 cells/ml) for the A549 cell line, which have a short doubling time. ASK and RTgill-W1 cells have lower rates of division, and for these cells we used a higher concentration (1000 000 and 1400 000 cells/ml, respectively). Meanwhile, too high or too low seeding densities can create problems, an example of which is presented in Fig. 4A and B.

A cell density too low will not provide an adequate foundation for observing cellular effects. On the other hand, when cell seeding density is too high this may potentially impair proliferation, lead to cell detachment and render the experimental results invalid. The duration of the experiment, i.e. proliferation time on the microscope slides before concluding the study, may also naturally impact the monolayer properties. Cell suspension should also be properly homogenized before pipetting onto slides, as previously expressed. If this step is not followed, this could also result in varying cell density between slides in a series. Furthermore, there is always the aspect that cells will grow differently in different areas on a surface. This could also result in certain areas on the slides with higher or lower densities.

In terms of histological staining, dehydration is a crucial step. When fixed cells are not completely dehydrated, water can create surface tension and the stain will not infiltrate properly [12,13]. From experience, this could result in a more transparent colour and brownish areas, as illustrated in Fig. 4C and D. Ensuring proper dehydration of cells is therefore important to produce more visually appealing and reliable results.

Lastly, the use of serum (here: FBS) in culture media must be considered with respect to downstream analyses. Serum proteins have been known to absorb certain dyes [12,14]. Through our observations we also see that serum proteins can take up colour through the histological staining process. It is therefore important to thoroughly wash the slides to remove all serum before staining.

In our method development, we have used cell lines with epithelial-like properties. From our observations, these grow well on glass surfaces. This may however not be the case for other types of cells. For instance, endothelial cells tend to grow poorly on non-coated surfaces [15]. Therefore, the type of microscopy slides used for experiments should be based on cell line properties. There are a number of specially designed microscope slides available, e.g. positively charged, or coated with poly-L-lysine, fibronectin, collagen, or gelatine. We encourage that future studies test different microscope slides with different cell lines using the described method.

## CRediT authorship contribution statement

**Kine Samset Hoem:** Methodology, Validation, Investigation, Writing – original draft, Writing – review & editing. **Hanne Lillerovde Ørstenvik:** Methodology, Validation, Investigation, Writing – original draft, Writing – review & editing. **Anne Elin Varhaugvik:** Investigation, Resources, Writing – review & editing. **Ann-Kristin Tveten:** Conceptualization, Supervision, Writing – review & editing.

## Declaration of competing interest

The authors declare that they have no known competing financial interests or personal relationships that could have appeared to influence the work reported in this paper.

## Data Availability

No data was used for the research described in the article.
